# Complex considerations for randomisation across linked randomised trials of complex interventions: illustration from the affinitie programme

**DOI:** 10.1186/1745-6215-16-S2-O32

**Published:** 2015-11-16

**Authors:** Robert Cicero, Rebecca Walwyn, Amanda Farrin, Robbie Foy, Jillian Francis, Natalie Gould, Fabiana Lorencatto, Simon Stanworth

**Affiliations:** 1 Leeds Institute of Clinical Trials Research, University of Leeds, Leeds, UK; 2 Leeds Institute of Health Sciences, University of Leeds, Leeds, UK; 3 School of Health Sciences, City University London, London, UK; 4 NHS Blood and Transplant, Oxford, UK

## 

Complex interventions are challenging to evaluate, as the same intervention can be used in a variety of settings. We will describe a large research programme that is developing and evaluating theory-based audit and feedback interventions to increase the uptake of evidence-based blood transfusion practice (AFFINITIE), encompassing two sequentially-linked factorial cluster-randomised trials, each evaluating the individual effect of two feedback interventions within national NHS Blood and Transplant audits, applied first to surgical use of blood and then to patients with haematological malignancies. This will enable us to have an understanding of the generalizability of the interventions across settings as part of a single research programme. As the two trials are national, each aiming to include 152 UK NHS Trust and Health Boards, we require most eligible clusters to take part in both trials. To minimise contamination and maintain equipoise, we require that clusters maintain their treatment allocation across trials. This poses new complications for the allocation of clusters to treatment arms, particularly for the second trial.

We will discuss how we accounted for the shift from sequential to all-in-one randomisation, outlining the key considerations during this process. We will describe the issues arising from randomisation occurring at two stages (once for each trial), including awareness of which clusters will drop out between the trials, which clusters will join for the second and what minimisation factors they have. We will explore the need to ensure the randomisation for the second trial is robust, either maintaining or recreating balance in the treatment allocations.

